# Dental Considerations in Children with Inherited Bleeding Disorders and Inhibitors: A Systematic Review

**DOI:** 10.3390/jcm13247743

**Published:** 2024-12-18

**Authors:** Sanja Vujkov, Branislav Bajkin, Duška Blagojević, Isidora Nešković, Jelena Komšić, Ana Tadić, Bojan Petrović

**Affiliations:** Faculty of Medicine, University of Novi Sad, 21000 Novi Sad, Serbia; sanja.vujkov@mf.uns.ac.rs (S.V.); branislav.bajkin@mf.uns.ac.rs (B.B.); duska.blagojevic@mf.uns.ac.rs (D.B.); isidora.neskovic@mf.uns.ac.rs (I.N.); jelena.komsic@mf.uns.ac.rs (J.K.); bojan.petrovic@mf.uns.ac.rs (B.P.)

**Keywords:** hemophilia A, hemostatics, blood coagulation disorders, inhibitors, oral hemorrhage, pediatric dentistry

## Abstract

**Background/Objectives**: This systematic review evaluates the effectiveness of various hematological treatment protocols and local hemostatic measures in preventing oral bleeding and other complications during and after dental treatments in children with inherited bleeding disorders and inhibitors. **Methods**: This study was registered in the PROSPERO database. The comprehensive search strategy for this systematic review was conducted across five databases, namely, PubMed, Google Scholar, Web of Science, Scopus, and Cochrane Library. The search was aimed at identifying relevant literature published from January 2000 up to February 2024. Eligible studies included those with various designs, such as randomized controlled trials (RCTs), observational studies, cohort studies, case–control studies, and cross-sectional studies. Data extraction was carried out systematically, and relevant information on study characteristics, interventions, treatment protocols, local measures, complications, and outcomes was collected. **Results**: The systematic review included a total of five studies, encompassing participants ranging from ages of 2 to 18 years. These studies varied in their scope, with some focusing on hemophilia A with inhibitors while others addressed broader inherited bleeding disorders. The interventions examined included various prophylactic and treatment measures such as Emicizumab, recombinant factor VIIa, and local hemostatic measures. The study outcomes primarily assessed the efficacy of these interventions in preventing postoperative bleeding and improving quality of life. Emicizumab has significantly shifted the treatment paradigm for children with inherited bleeding disorders and inhibitors. This prophylactic treatment has been associated with a marked reduction in the frequency of bleeding episodes, fewer hospital admissions for bleeding management, and enhanced participation in daily activities. **Conclusions**: This review highlights gaps in the management of dental care in children with inherited bleeding disorders and inhibitors. It underscores the need for standardized protocols that integrate new prophylactic treatments such as Emicizumab. Our findings suggest that adopting updated protocols can significantly reduce bleeding complications during dental procedures.

## 1. Introduction

Inherited bleeding disorders (IBDs) are genetic conditions that cause abnormalities in blood clotting, leading to an increased risk of excessive bleeding. Common IBDs include hemophilia A (HA), hemophilia B (HB), von Willebrand disease (VWD), and other rare factor deficiencies. Individuals with IBD often experience prolonged bleeding following injuries, medical or dental procedures, and spontaneous bleeding into joints and soft tissues [1,2,3,4].

The development of inhibitors is the most serious treatment-related complication in patients with congenital bleeding disorders. Inhibitors are antibodies that neutralize the function of infused clotting factors, particularly factors VIII (FVIII) and IX (FIX) in hemophilia, making it challenging to control bleeding. This complication occurs in approximately 30% of patients with severe HA and 5–10% of those with mild to moderate forms. In HB, inhibitor formation occurs in up to 5% of patients, often associated with allergic reactions to FIX in about 50% of cases. Inhibitor formation is a serious complication of replacement therapy in type 3 VWD, with a prevalence of 7.5%. About 10% of the patients who develop inhibitors show severe anaphylactic reactions to any products containing VWF and they become non-responsive. Inhibitor development is influenced by factors such as gene mutation, family history, and treatment variables, but the underlying mechanisms are not fully understood [1,5,6].

Managing bleeding in patients with inhibitors has become increasingly sophisticated. Strategies include immune tolerance induction (ITI), where repeated doses of clotting factors are administered to reduce or eliminate the immune system’s response, alongside bypassing agents like activated prothrombin complex concentrate (aPCC) and recombinant factor VIIa (rFVIIa). Novel therapies such as recombinant porcine FVIII (rpFVIII), bispecific monoclonal antibodies (e.g., Emicizumab), and small interfering RNAs (siRNAs) also play a role [7,8]. These advancements have notably enhanced bleed prevention and overall quality of life for patients with inherited bleeding disorders (IBDs) [7,8]. The World Federation of Hemophilia (WFH) recommends specific protocols for patients with hemophilia A (HA) and inhibitors for those undergoing surgeries or invasive procedures, including higher doses of FVIII for low-responding inhibitors and bypassing agents for high-responding inhibitors. Those on Emicizumab prophylaxis are recommended rFVIIa, with combination therapies being closely monitored in specialized centers to avoid thrombotic complications [1,9,10,11,12,13].

Dental care represents a critical component of comprehensive healthcare for persons with IBD, particularly those with inhibitors. Dental procedures, such as local anesthesia, tooth extractions, or oral surgeries, pose significant bleeding risks. Dental practitioners must be knowledgeable about these complications and the associated treatment options, tailoring care to ensure optimal outcomes [14,15]. Preventive dental management is crucial to minimize the need for coagulation factor cover during procedures [15]. Recent advancements have altered the protocols for minor surgeries and dental extractions, yet data on pediatric patients with rare bleeding disorders or inhibitors remain sparse [16,17,18,19].

Living with an IBD, especially in the presence of inhibitors, profoundly impacts the quality of life for children and their families, underscoring the need for comprehensive psychological, social, and medical support. Optimal care for people with hemophilia, particularly those with severe forms, requires a multidisciplinary team, including dentists, to ensure the best health and quality of life outcomes. Over the last few decades, treatment options for patients with IBD have significantly improved, especially with the use of prophylactic approaches, enhancing both a positive prognosis and daily life [2,3,4,5].

The rationale for conducting this systematic review stems from the observed variability in treatment outcomes for children with inherited bleeding disorders undergoing dental procedures. Despite advancements in medical and dental care, significant gaps remain in the evidence-based management of these patients, particularly those with inhibitors. This review aims to consolidate the current knowledge, identify effective treatment protocols, and highlight areas where further research is needed. The scientific value of this review lies in its potential to influence clinical guidelines and improve outcomes for a vulnerable patient population, thereby addressing an urgent need within pediatric hematology and dental care, particularly tooth extractions, in children with IBD and inhibitors.

## 2. Materials and Methods

### 2.1. Study Design

This study was conducted as a systematic review. The aim was to comprehensively assess the effectiveness of various treatment protocols and local hemostatic measures in children with inherited bleeding disorders and inhibitors. The systematic review was conducted to evaluate the effectiveness of various treatment protocols and local hemostatic measures in children with inherited bleeding disorders and inhibitors. The review was structured in accordance with the Preferred Reporting Items for Systematic Reviews (PRISMA) guidelines, ensuring transparency and methodological rigor. The study has been registered on the International Prospective Register of Systematic Reviews (PROSPERO) database under the number CRD42024502409.

### 2.2. Eligibility Criteria

Inclusion criteria for article selection were full-text articles published in English in peer-reviewed journals; those published from 2000 up to February 2024; randomized controlled trials (RCTs), non-randomized controlled trials, cohort studies, case–control studies, case reports, cross-sectional studies, and observational studies with comparative designs; and single-center and multi-center studies. The eligibility criteria for patients’ inclusion in this systematic review were as follows: children and adolescents under the age of 18, diagnosed with IBD and inhibitors; dental treatment, extractions, endodontic therapy and preventive measures. If applicable, comparisons could be made between different dental care approaches, types of bleeding disorders, age groups, or types of dental interventions. The impact of inhibitors on dental care and potential differences in outcomes based on the type of bleeding disorder or other relevant factors should be considered. Exclusion criteria for studies were as follows: abstracts, posters, conference abstracts, dissertations, and unpublished studies; duplicate studies or overlapping datasets and studies with insufficient data or incomplete reporting.

### 2.3. Sources of Information and Search Strategy

The search strategy was carried out in February, 2024, and included five databases: PubMed, Google Scholar, Web of Science (WOS), Scopus and Cochrane Reviews. The key words used for the search were “hemophilia” OR “haemophilia” AND “bleeding disorders” AND “inhibitors”, “oral health” and “oral bleeding”. The search terms were applied to the title and abstract fields of the articles. The search strategy aimed to identify relevant studies evaluating the dental considerations in children with IBD and inhibitors. To systematically extract pertinent data from the listed research, structured data extraction templates were created. Microsoft Excel spreadsheet software (Version 2411, Build 18227.2016) was used to develop these templates. The forms had pre-set fields for important study characteristics, results, and other pertinent information. All review-related documents and data were put in a safe, centralized location. This was accomplished using Google Drive, a cloud-based storage service. All review operations, including search tactics, screening procedures, data extraction techniques, and any adjustments or choices made throughout the study, were meticulously documented.

### 2.4. Study Selection

Multiple phases of the study selection procedure were carried out by two independent reviewers (SV and BP). During the screening and data extraction phases, any disagreements between reviewers were initially discussed in an attempt to reach a consensus. If a consensus could not be achieved through discussion, the issue was escalated to a third, senior reviewer (BB) whose expertise in the field was used to make a final decision. Each study was thoroughly reviewed based on the stated eligibility requirements. Regarding their applicability to the specific goals of the review as well as the research issue, the included studies underwent a comprehensive evaluation. The final study selection process was documented.

### 2.5. Data Collection

Articles that satisfied the inclusion criteria were screened, and the extraction process was carried out in a controlled and systematic way. The authors, the publication year, and the country where the study was conducted were taken out of each included paper. The collected data were analyzed regarding type of the study, type of IBD with inhibitors, hematological treatment, local hemostatic measures, and dental treatment. To ensure consistency in data extraction across all included studies, we employed a standardized extraction form designed to comprehensively capture essential information such as study design, population demographics, interventions, outcomes, and any noted complications. All reviewers were familiar with the proper use of this form to minimize variability in data interpretation and entry.

## 3. Results

### 3.1. Study Selection

Our comprehensive search strategy targeted five key databases: PubMed, Scopus, Google Scholar, Web of Science, and Cochrane Reviews. The initial search yielded a total of 9402 references, reflecting the extensive literature available on the topic.

#### 3.1.1. Initial Filtering

Duplications Removed: We first eliminated 5374 duplicate entries to ensure each study was only counted once.

Non-English Articles: A further 273 records were excluded because they were not in English, aligning with our language criteria for inclusion.

Non-Research Articles: An additional 300 records were identified and removed because they were not research articles (e.g., opinion pieces and editorials).

#### 3.1.2. Title and Abstract Screening

After refining our dataset, 3455 articles underwent a thorough review of their titles and abstracts. This process led to the exclusion of 3000 records due to irrelevance to the study criteria, such as studies focusing on adult populations or treatments unrelated to inhibitors in pediatric patients.

#### 3.1.3. Full-Text Review

Of the remaining 455 articles, we encountered access issues with 345, leaving 110 articles available for full-text review.

Following detailed evaluation, 105 of these were further excluded due to various reasons including inadequate methodology, incorrect study design, or incomplete data reporting.

#### 3.1.4. Studies Included

**Figure 1:**
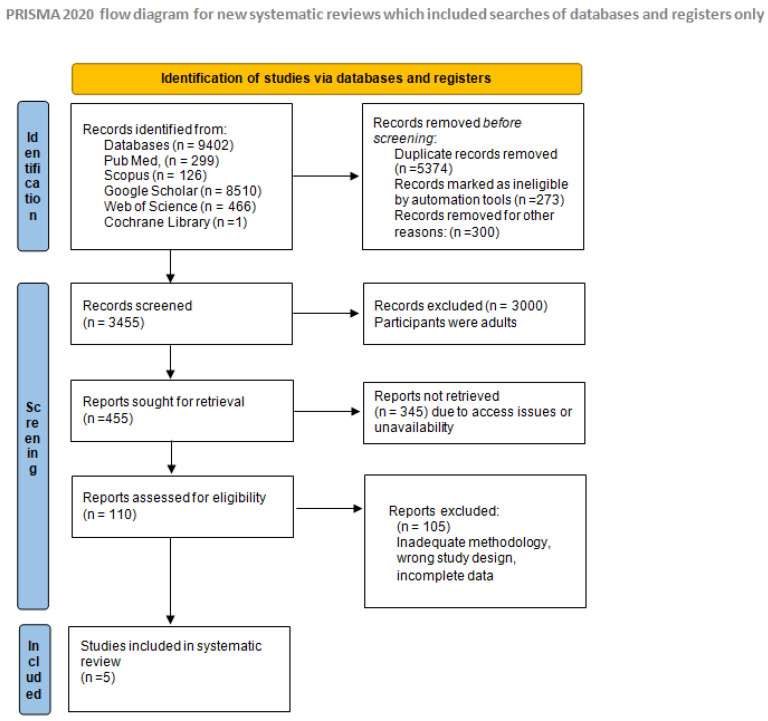
PRISMA flow diagram summarizing study selection.

Ultimately, five publications fully met our inclusion criteria and were selected for a detailed analysis in this systematic review, as shown in Figure 1. These studies provided valuable insights into dental treatments and associated complications in children with inherited bleeding disorders and inhibitors.

This figure provides a visual representation of the search and selection process used in our systematic review, detailing the number of studies screened, assessed for eligibility, and included in the final analysis.

Table 1 represents summary of included studies.

This table details the patient demographics, specific dental interventions, and outcomes of the studies included in our review, providing a comprehensive overview of the data analyzed and the context of the findings.

### 3.2. Overview of Included Studies

Table 1 summarizes the systematic review, which encompassed five studies documenting dental interventions and oral hemorrhage outcomes in pediatric patients afflicted with inherited bleeding disorders (IBDs) and inhibitors. These studies collectively analyzed a total of 13 patients, including 11 diagnosed with severe hemophilia A (HA) and 2 with type 3 von Willebrand disease (VWD), all of whom had developed inhibitors. The five included studies detailed dental interventions and outcomes for a cohort of 13 pediatric patients with inherited bleeding disorders and inhibitors. The characteristics of these studies are summarized below:

- Population: Included a total of 13 patients, with 11 having severe hemophilia A (HA) and 2 with type 3 von Willebrand disease (VWD), all with inhibitors.
- Interventions: The studies predominantly involved tooth extractions (five primary and eight permanent, including two surgical), alongside other dental procedures like root canal treatments and management of gingival hemorrhage.
- Outcomes: The primary outcome focused on was postoperative bleeding. Four out of seven patients in one study experienced prolonged bleeding, requiring additional interventions, whereas other studies reported successful management with no postoperative complications.
- Treatments Used: Systemic therapies such as recombinant activated factor VII (rFVIIa) and Emicizumab were commonly employed, complemented by local hemostatic measures like gelatin sponge, fibrin glue, and oral antifibrinolytic agents (OAFAs).

### 3.3. Dental Interventions and Therapeutic Measures

The primary dental interventions across these studies included the extraction of five primary and eight permanent teeth, with two cases involving surgical extractions. Additional treatments reported included root canal treatments, management of gingival hemorrhage, treatment for traumatic oral bleeding, and interventions for bleeding associated with tooth eruption. Systemic therapies commonly employed were rFVIIa and Emicizumab, used alongside local hemostatic measures such as gelatin sponge, fibrin glue, and OAFAs. For patients with VWD, plasma-derived von Willebrand factor concentrate (pdvWF) was used to manage gum bleeding and post-extraction bleeding.

### 3.4. Outcomes and Efficacy of Interventions

The primary outcomes centered on the effectiveness of the interventions in managing postoperative bleeding. Key findings include the following:

- Reduction in Postoperative Bleeding: The studies consistently reported a reduction in postoperative bleeding among patients treated with Emicizumab, showcasing its effectiveness as a prophylactic measure.
- Successful Management with rFVIIa: The use of recombinant activated factor VII (rFVIIa) was effective in achieving hemostasis in cases with prolonged bleeding episodes, indicating its critical role in emergency interventions.
- Enhanced Safety Profiles: The combination of systemic therapies and local hemostatic measures resulted in fewer hospitalizations and complications, underscoring the safety and efficacy of these treatment protocols.

### 3.5. Trends and Contrasts Across Studies

The review identified several key trends and contrasts that highlight the variable impact of different interventions:

- Trend in Using Emicizumab: A noticeable trend across studies was the successful use of Emicizumab in reducing postoperative bleeding, especially notable in studies by Young, McCary, and Yagyuu. This contrasts with older therapies that showed varied success rates.
- Contrast in Outcomes Based on Treatment Protocols: Studies like Laguna’s showed that when traditional treatments such as rFVIIa were used alone, there was a higher incidence of prolonged bleeding, necessitating additional interventions. In contrast, the combination of Emicizumab with local hemostatic measures generally led to better outcomes, highlighting the importance of comprehensive treatment strategies.
- Variability in Response Based on Inhibitor Severity: The severity of inhibitors significantly influenced outcomes, with studies reporting that patients with higher inhibitor titers faced more complications. This variability underscores the need for personalized treatment approaches tailored to the inhibitor profile of each patient.

### 3.6. Impact of Emicizumab and Hemostatic Interventions

- Reduction in Hospitalizations: The studies included in our review consistently reported a reduction in the need for hospitalizations due to complications from dental procedures in patients treated with Emicizumab. This finding highlights the drug’s effectiveness in managing bleeding risks associated with dental work in pediatric patients with inhibitors.
- Enhanced Control of Bleeding: Emicizumab, when used in conjunction with traditional hemostatic measures such as gelatin sponges, fibrin glue, and oral antifibrinolytic agents, was shown to significantly reduce the frequency and severity of postoperative bleeding episodes. This synergy between systemic and local therapies provides a robust defense against the unique challenges faced by these patients.
- Clinical Significance: These interventions not only improve patient outcomes but also contribute to more sustainable healthcare practices by reducing the overall burden on hospital resources. The evidence suggests that integrating these therapies into standard treatment protocols could greatly enhance the quality of life for these vulnerable populations.

In assessing the included studies, we employed standardized quality and bias assessment tools, finding a range of evidence levels (II to IV) and identifying potential selection and reporting biases, which underscore the need for the careful interpretation and application of our findings in clinical practice.

## 4. Discussion

### 4.1. Key Findings

The systematic review identified key interventions that significantly improve the management of dental care in pediatric patients with inherited bleeding disorders and inhibitors. Our analysis revealed that the prophylactic use of Emicizumab, combined with traditional hemostatic measures, substantially reduces the incidence of postoperative bleeding and the need for hospitalizations, thereby enhancing patient outcomes and healthcare efficiency.

### 4.2. Clinical Implications

This systematic review synthesizes the evidence on the effectiveness of various interventions, including Emicizumab, in managing dental procedures in pediatric patients with inherited bleeding disorders and inhibitors. Our findings demonstrate a significant reduction in postoperative bleeding and hospitalization rates, supporting Emicizumab’s role as a transformative agent in clinical practice. The prophylactic use of Emicizumab represents a substantial advancement in the clinical management of bleeding disorders. By significantly reducing the frequency and severity of bleeding episodes, Emicizumab not only improves patient outcomes but also contributes to more efficient healthcare resource utilization. Its integration into routine care protocols could redefine standard practices, emphasizing a preventative approach that could shift the paradigm in pediatric hematology. Future research should focus on longitudinal studies to further validate Emicizumab’s long-term benefits and investigate any potential risks or limitations associated with its use in younger populations. Additionally, exploring its integration into broader treatment protocols will be crucial for establishing comprehensive care guidelines that can be universally recommended.

Dental treatment and tooth extraction in children with IBD and inhibitors is always a high-risk procedure, which often presents problems associated with bleeding. The review ascertained that the implementation of prophylactic Emicizumab, in conjunction with rFVIIa and local hemostatic agents such as gelatin sponge and fibrin glue, demonstrated particular effectiveness in reducing postoperative hemorrhage and the necessity for hospitalization [11,12,13]. Although a subset of patients exhibiting elevated inhibitor levels encountered protracted bleeding, the majority of cases managed with these advanced protocols attained successful hemostasis. These outcomes accentuate the critical significance of personalized treatment regimens and the notable progressions in the management of dental care for this at-risk pediatric population [23,24].

The results of this review are congruent with the existing body of literature, which consistently underscores the difficulties associated with the management of dental procedures in pediatric patients exhibiting IBD and inhibitors. Prior research has similarly documented the efficacy of rFVIIa and antifibrinolytic agents alongside a variety of local hemostatic agents in facilitating hemostasis during dental interventions [14,15,16,17]. Windya [25] presented the concomitant use of aPCC and TXA as a standard of care in patients with IBD and inhibitors who suffer from mucosal bleeds or undergo invasive procedures on mucosal membranes (e.g., dental extraction). Nevertheless, significant inconsistency is noted regarding the degree of postoperative hemorrhage, which seemingly correlates with the specific levels of inhibitors and the nature of the dental procedures performed [26]. Several investigations have indicated elevated incidences of prolonged bleeding, which could be ascribed to disparities in patient demographics, fluctuations in inhibitor titers, or less uniform treatment protocols [27,28].

Recent advancements, particularly the advent of Emicizumab as a prophylactic option, have markedly transformed the paradigm of dental care for individuals diagnosed with IBD and inhibitors [29]. Emicizumab offers a consistent and enduring safeguard against bleeding, even in patients possessing high-titer inhibitors. This review substantiates that the application of Emicizumab, in conjunction with rFVIIa and local hemostatic measures, diminishes both the frequency and severity of postoperative bleeding, consistent with recent research that elucidates its contribution to enhancing clinical outcomes and alleviating the burden of care. The transition towards protocols incorporating Emicizumab signifies a substantial progression in the management of these complex cases, providing more reliable and safer approaches for dental interventions [9,10,11].

The results derived from this systematic review bear considerable implications for the field of dental practice, particularly concerning the management of pediatric patients afflicted with IBD and inhibitors. The evidenced efficacy of Emicizumab and rFVIIa in preventing bleeding occurrences during and subsequent to dental procedures emphasizes the necessity of integrating these therapeutic modalities into clinical protocols [13,14]. For dental practitioners, this necessitates the adoption of a more anticipatory strategy in treatment planning, wherein personalized care is of the utmost importance. Through the meticulous evaluation of each patient’s distinct inhibitor levels, bleeding history, and overall health status, dental professionals can customize their treatment approaches to minimize the likelihood of adverse complications. The implementation of local hemostatic interventions, such as gelatin sponges and fibrin glue, alongside systemic therapies, ought to be institutionalized as an integral component of comprehensive care regimens for these patients at heightened risk [16,17].

### 4.3. Challenges

However, the dental management in individuals with IBD and inhibitors introduces specific challenges that necessitate thorough consideration. The heterogeneity in inhibitor levels, the complexity of dental procedures, and the complete unpredictability of bleeding episodes complicate the standardization of treatment protocols [27]. Moreover, the imperative for interdisciplinary collaboration is crucial, as favorable outcomes frequently hinge upon effective communication among dental professionals, hematologists, and other healthcare practitioners. The coordination of care across various specialties guarantees that all dimensions of the patient’s condition are comprehensively addressed, spanning from preoperative strategizing to postoperative monitoring [26]. The potential for complications, such as extended bleeding or the emergence of thrombotic incidents, further accentuates the necessity for scrupulous planning and the provision of specialized resources within dental environments [24,27]. Overcoming these challenges necessitates continuous education and training for dental practitioners, alongside the formulation of explicit guidelines to support evidence-based practice.

### 4.4. Limitations

Our systematic review, while providing valuable insights, encounters several limitations that could impact the interpretation and generalization of the findings. We categorize these into study-level and review-level limitations for clarity.

Study-Level Limitations:

- Sample Size and Population Diversity: The small number of studies and the limited diversity within study populations may affect the generalizability of our results. Smaller sample sizes limit the statistical power to detect smaller effects, which might lead to an underestimation or overestimation of the treatment effects.
- Heterogeneity in Study Designs: Variations in study design, including differences in intervention types and outcome measures, contribute to data heterogeneity. This makes it challenging to perform a meta-analysis and could obscure potential differences or similarities between studies.

Review-Level Limitations:

- Publication Bias: There is a potential for publication bias, as studies showing positive results are more likely to be published than those with negative or inconclusive results. This bias could lead to an overrepresentation of positive findings in our review.
- Language Restrictions: By excluding non-English studies, we may have omitted relevant research, limiting the comprehensiveness of our review and potentially biasing our conclusions towards findings predominantly from English-speaking countries.

Implications of Limitations:

These limitations suggest that while our findings provide significant insights into the effectiveness of interventions like Emicizumab, they should be interpreted with caution. The potential biases and variability in study design necessitate further research with broader and more diverse populations to confirm these findings and expand their applicability.

### 4.5. Future Directions

The outcomes of this review highlight considerable gaps within the extant literature, emphasizing the necessity for additional inquiry to enhance the comprehension of optimal dental management for pediatric patients afflicted with IBD and inhibitors. A particularly urgent domain for forthcoming research pertains to the imperative for larger, multicenter investigations that could yield more definitive directives regarding treatment protocols. Such investigations ought to aspire to standardize the application of systemic interventions, including Emicizumab and rFVIIa, alongside local hemostatic strategies, to ascertain the most efficacious combinations and dosages for mitigating bleeding during and subsequent to dental interventions [8,25,26]. Moreover, scholarly efforts should concentrate on the longitudinal outcomes associated with these treatments, specifically regarding quality of life, the incidence of thrombotic complications, and the overall safety profile within pediatric cohorts [30,31].

The potential of novel and emerging therapeutic modalities presents promising avenues for enhancing dental care for these patients. Innovations such as gene therapy, small interfering RNAs (siRNAs), and other avant-garde hemostatic agents are presently in clinical trials and possess the potential to transform the management of bleeding disorders [5]. The incorporation of these therapies into dental care frameworks could further diminish the risks inherent in dental procedures for children with IBD and inhibitors, providing more effective and less invasive alternatives. Future research should focus on the assessment of the efficacy and safety of these emerging interventions within the realm of dental care, with a focus on formulating comprehensive, evidence-based guidelines that can be extensively implemented in clinical settings.

## 5. Conclusions

This systematic review has demonstrated the critical importance of customized treatment protocols in managing dental care for pediatric patients with inherited bleeding disorders (IBDs) and associated inhibitors. Key findings affirm the effectiveness of therapeutic interventions, particularly Emicizumab and recombinant activated factor VII (rFVIIa), in mitigating bleeding complications during and post dental procedures.

**Clinical Implications:** These results have direct implications for clinical practice, advocating for the adoption of these interventions as standard care to enhance patient safety and outcomes. The evidence strongly supports the integration of Emicizumab into routine prophylactic strategies, significantly reducing the frequency and severity of bleeding episodes, thereby minimizing the need for acute care and potential hospitalizations.

**Gaps and Future Directions:** Despite these advances, the review identifies critical gaps in the literature, notably, the lack of long-term outcome studies and comprehensive quality of life assessments. These areas require further exploration to fully understand the long-term benefits and potential risks associated with these treatments. Additionally, the development and validation of standardized treatment guidelines across diverse clinical settings remain essential to ensure consistent and effective care.

**Recommendations for Future Research:** Future research should expand to include cost-effectiveness analyses to evaluate the feasibility of the widespread adoption of novel therapies like Emicizumab. Studies should also explore the integration of emerging therapies and their impact on treatment paradigms, particularly in resource-limited settings.

**Interdisciplinary Collaboration:** The effective management of IBD in pediatric patients necessitates a collaborative approach among various healthcare providers. Strategies should include regular consultations between dentists, hematologists, and pediatricians to coordinate care and optimize treatment outcomes. Training programs and joint clinical sessions could be instrumental in fostering this interdisciplinary collaboration, ensuring all practitioners are well versed in the complexities of treating patients with severe bleeding disorders.

## Figures and Tables

**Table 1:** Dental treatment in children with IBD and inhibitors.

Author	Study Design	Systemic Treatment	Local Hemostatic Measures	Number of Patients Age	Type of Dental Treatment	Type of IBD, Level of Inhibitors	Postoperative Bleeding	Comment
Laguna, 2005. Poland [19]	CC, SC	**rVIIa** 90–100 μg/kg **OAFA** 20 mg/kg every 6 h **FEIBA**	gelatin sponge, fibrin glue	*n* = 5 M 8–13 years	−7 ex (4 primary and 3 permanent teeth), -oral bleeding during tooth eruption	HA, S high titers of inhibitor ranging from 15 to 958 BU/mL	4/7 prolonged bleeding 1. 6 h post-ex, rVIIa × 3, fibrin glue 2. post-ex, rVIIa 3. 2 weeks of bleeding during tooth eruption; rFVIIa and FEIBA. Anemia, blood transfusions, 5 times. rVIIa secured hemostasias (three doses) 4. post-ex bleeding; 7 doses of rVIIa, gelatin sponge	4 inpatient/1 outpatient
Young [20] 2019	P, MC	**Emicizumab** 1.5 mg/kg 1× weekly prophylaxis		*n* = 2 M <18 y	2 traumatic mouth bleeds	HA, inhibitors	rVIIa 1 dose	2/2 inpatients
McCary [21] 2020 USA	O, MC	**Emicizumab** 3 mg/kg weekly, 4 dozes; followed by weekly, every other week or monthly		*n* = 2 M 9 y, 8 y	2 dental procedures 1. root canal tr 2. primary tooth ex	HA, inhibitors	0/2 1. AA 2. pdvWF 66 AA	no complications
Jazebi [22] 2021 Iran	R, SC	pdvWF on-demand Haemate		*n* = 2 10 y F, <18 y M	2 oral bleedings 1. gum bleeding 2. post-dental ex bleeding	VWD type 3, inhibitors		
Yagyuu, 2023 Japan [18]	CS, O, SC	**Emicizumab** 1.5 mg/kg once weekly **rFVIIa** 90 μg/kg 1 h before ex and 3–6 h post ex **OAFA** 20–25 mg/kg	gelatin sponge, oxidized cellulose, and/or fibrin glue	*n* = 2 M <18 y	4 ex (2 surgical + 2 ex)	HA, S inhibitors	0/4	inpatients, no complications

Note: R—retrospective; P—prospective; Cs—case series; SC—single center; MC—multicenter; CC—case–control; O—observational; rVIIa—recombinant activated factor VII; pdvWF—plasma-derived von Willebrand concentrate; Haemate—von Willebrand concentrate/factor VII; AA—Aminocaproic acid; FEIBA—factor VIII inhibitor bypassing activity; OAFA—oral anti-fibrinolytic agent; M—male; F—female; HA—hemophilia A; VWD—von Willebrand disease; S—severe; ex—tooth extraction.
